# Emerging Paradigms in Cancer Metastasis: Ghost Mitochondria, Vasculogenic Mimicry, and Polyploid Giant Cancer Cells

**DOI:** 10.3390/cancers16203539

**Published:** 2024-10-19

**Authors:** Mateusz Krotofil, Maciej Tota, Jakub Siednienko, Piotr Donizy

**Affiliations:** 1 Department of Clinical and Experimental Pathology, Wroclaw Medical University, Borowska 213, 50-556 Wroclaw, Poland; 2 Department of Experimental Oncology, Ludwik Hirszfeld Institute of Immunology and Experimental Therapy, Polish Academy of Sciences, 53-114 Wroclaw, Poland; 3 Department of Pathology and Clinical Cytology, Jan Mikulicz-Radecki University Hospital, 50-556 Wroclaw, Poland

**Keywords:** metastasis, cancer, ghost mitochondria, vasculogenic mimicry, polyploid giant cancer cells, GM, VM, PGCCs

## Abstract

The capacity of cancer cells to migrate from a primary tumor, disseminate throughout the body, and eventually establish secondary tumors is a fundamental aspect of metastasis. A detailed understanding of the cellular and molecular mechanisms underpinning this multifaceted process would facilitate the rational development of therapies aimed at treating metastatic disease. Although various hypotheses and models have been proposed, no single concept fully explains the mechanism of metastasis or integrates all observations and experimental findings. Recent advancements in metastasis research have refined existing theories and introduced new ones. This review evaluates several novel/emerging theories, focusing on ghost mitochondria (GM), vasculogenic mimicry (VM), and polyploid giant cancer cells (PGCCs).

## Simple Summary

Metastatic disease is the leading cause of cancer-related morbidity and mortality, accounting for over 80% of cancer deaths. Recent studies have introduced and refined several theories on cancer metastasis, including ghost mitochondria (GM), vasculogenic mimicry (VM), and the formation of polyploid giant cancer cells (PGCCs). Understanding the complex processes of cancer metastasis is crucial for developing effective treatment options.

## 1. Introduction

Metastasis is a complex process in cancer biology that is not fully understood. Disseminated cancer is often a fatal disease and represents a significant global health challenge [1,2,3]. Among a cohort of 1,030,937 metastatic cancer survivors in the United States from the period 1992–2019, 82.6% of the patients (*n* = 688,529) died from the cancer for which they were diagnosed. The median survival duration for patients living with metastases is 10 months [4]. The primary hypothesis explaining the formation of nodal and distant organ metastases is based on direct invasion by tumor cells into lymphatic and/or blood vessels and the generation of circulating tumor cells (CTCs) within the lymphatic and blood vessel systems [5,6]. However, the process of CTC colonization of regional lymphatic nodes and distant organs seems to be disproportionately insufficient [7,8,9]. Studies on metastasis in patients with melanoma have shown that only 2.5% of circulating cancer cells are able to survive and form micrometastases [9]. Moreover, the formation of micrometastases does not always result in the development of metastatic tumors, as only 1% of micrometastases progress to disseminated disease [9]. These discoveries are inconsistent with the clinically observed high efficiency of the metastatic process, which occurs even in patients who undergo aggressive treatment [10,11]. In our work, we review the literature to reveal potential novel concepts related to cancer cell metastasis.

## 2. “Ghost” Mitochondria (GM)—Cancer Cell Communication or Independent Metastatic Route?

The role of mitochondria in cancer was first identified by Otto Warburg, who hypothesized that there is a mitochondrial defect in cancer cells [12]. Research has shown that this defect should be considered not only an important factor of cancer cell metabolism but also an essential part of carcinogenesis and metastasis. Thomas Seyfried proposed that initial changes in cancer cells are not associated with mutations in genes but with defective mitochondrial metabolism [13]. His hypothesis was based on numerous in vitro studies that described the suppression of carcinogenesis in hybrid tumor cells after the transplantation of normal cell cytoplasm or mitochondria [14,15,16,17]. Other studies revealed that the transfer of mtDNA from cells with high metastatic potential to cells with low metastatic potential can generate high metastatic potential in recipient cells [18].

Mitochondria in cancer cells exhibit remarkable adaptability to endure adverse conditions, including hypoxia, nutrient deficiency, and various therapeutics. Consequently, they are integral to tumor development, requiring a capacity for adjustment in response to both cellular and environmental fluctuations. In addition to their role in bioenergetics, numerous other facets of mitochondrial biology are associated with cellular transformation. These processes encompass mitochondrial biogenesis and turnover, metabolic pathways, dynamics of fission and fusion, regulation of oxidative stress, susceptibility to cell death, and signaling mechanisms [19].

Cancer cells are able to produce various types of microparticles containing extracellular vesicles (EVs) surrounded by a lipid bilayer and nonmembranous particles [20,21,22]. EVs can be divided into large exopheres, which can contain cellular organelles, and smaller microvesicles (>150 nm) and exosomes (30–150 nm) [22,23]. EVs containing mitochondria can not only appear locally but also be released into the bloodstream by both healthy and injured cells [24,25,26]. The number of excreted mitochondria is elevated in injured cells, including cancer cells [24,26]. Additionally, cancer cells release small exosomes that contain mitochondrial proteins or mtDNA. These exosomes are present in blood and may serve as potential new cancer markers detectable in blood samples [27,28]. Microparticles that carry mitochondria often present surface markers of platelets or immune cells to promote inflammation [26,29]. Circulating mitochondria are involved in immune response modulation and are important in intracellular signaling pathways [30]. Cancer cells can receive healthy mitochondria from tumor microenvironment (TME) cells, which may have a therapeutic effect and suppress tumor growth [14,31,32]. However, mitochondrial transfer can also enhance cancer cell metabolism and is associated with progression and metastasis [33,34]. Studies of mitochondrial transfer in the TME revealed that prostate cancer cells can recruit mitochondria from stromal fibroblasts [21]. Cancer cells can receive mitochondria from tumor-associated stromal cells even if functional mitochondria are already present in their cytoplasm. The process of mitochondrial transplantation between cells is locally regulated. Only fibroblasts reprogrammed by cancer cells to be highly glycolytic can transfer their mitochondria, and only selected cancer cells can act as recipients [21]. The authors suggested that the receiving mechanisms may depend on lactate excreted by cancer-associated fibroblasts, which triggers changes in cancer cell metabolism and activates signaling pathways associated with mitochondrial uptake [21]. Mitochondrial transplantation is vital for cancer cells to produce sufficient energy for active proliferation [21,32,35]. Studies of cancer mitochondrial transfer are consistent with discoveries claiming that functional mitochondria and oxidative energy production are essential for tumor cell proliferation [24,26,36,37]. Alterations in energy metabolism or the function of mitochondria may inhibit the anticancer capabilities of immune cells and promote the immune escape of cancer cells within the TME [38,39]. Recent research suggests that cancer cells may have the ability to appropriate mitochondria from non-tumor cells within the TME [40]. Mitochondrial transfer is considered a form of intercellular communication. It has been posited that cancer cells could acquire mitochondria from T cells through nanotubes, thereby enhancing their own cellular strength and facilitating immune escape.

Interestingly, mitochondrial transfer by platelets is performed in different physiological and pathological states, e.g., wound healing. Activated platelets release mitochondria to fibroblasts in wounds to enhance metabolic processes and promote angiogenesis [41]. Studies have revealed that mitochondrial transplantation between platelets occurs in cancer and can potentially be associated with metastasis and proliferation. Zhang et al. [42] reported that osteosarcoma cells are reprogrammed into a metastatic state through the acquisition of platelet mitochondria via the PINK1/Parkin-Mfn2 pathway. Platelet mitochondria promote lung metastasis by regulating the GSH/GSSG ratio and reactive oxygen species (ROS) in cancer cells. Mitochondrial transfer between cells possibly plays an important role in local intracellular interactions and is potentially a novel target in cancer therapy.

However, cancer cells contain not only functional but also damaged and nonfunctional cancer mitochondria called “ghost” mitochondria (GM) [43]. The term GM refers to spherical mitochondria that arise from the downregulation of Mic60 in cancer cells [44]. Mic60 is an essential component of the mitochondrial contact site and cristae organizing system (MICOS), which is pivotal for the development of the inner mitochondrial membrane and the formation of functional cristae [45]. Impaired MICOS function results in the formation of mitochondria, which are unable to fulfil their major functions: effective ATP production and the regulation of cell death [43,45]. The presence of the GM may seem contradictory to the energy demands of cancer cells, but it is linked to several proinflammatory signaling pathways, the regulation of nuclear gene expression, and the evasion of programmed cell death mechanisms [43]. Moreover, the presence of the GM can possibly influence cancer metastasis because there seems to be a connection between the downregulation of Mic60 and the ability of cells to produce metastases [43].

Oxidative phosphorylation (OXPHOS) significantly contributes to the advancement of various cancer cells. It not only supplies adequate energy essential for the survival of tumor tissue but also modulates the conditions necessary for tumor proliferation, invasion, and metastasis. Furthermore, modifications in OXPHOS can adversely affect the immune functionality of immune cells within the TME, resulting in immune escape. Mic60-low tumors demonstrate a significant decline in mitochondrial functionality, which is paradoxically correlated with an enhanced tendency for metastasis. An elevated expression of the Mic60-low gene signature has been linked to reduced patient survival in various cancer types, including kidney cancer, uveal melanoma, testicular germ cell tumors, and thymomas [46].

**Figure 1:**
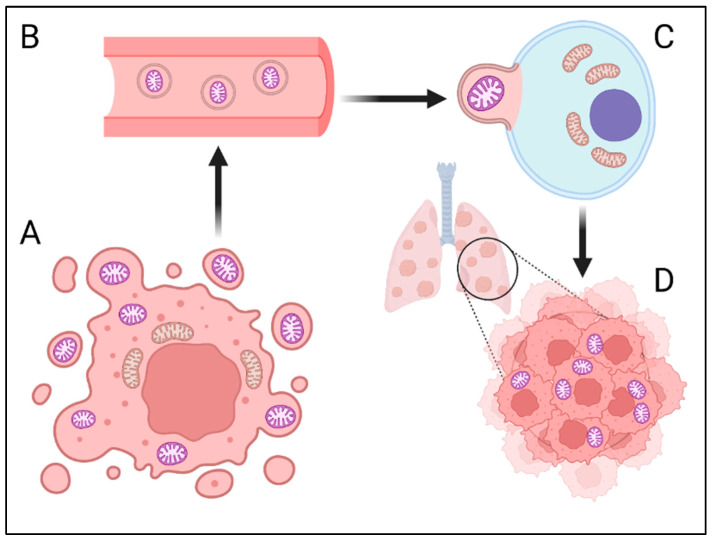
The possible role of circulating “ghost” mitochondria (GM) in metastasis. (**A**) The GM is released by cancer cells into the tumor microenvironment (TME). Vesicles with mitochondria act as local signaling factors but can also enter the circulation. (**B**) Circulating GM enters sites of cancer metastasis through the bloodstream. (**C**) Vesicles with a GM fuse with the membrane of niche cells and modify their metabolism and gene expression to create a TME that is optimal for metastasis. (**D**) Previously healthy cells with internalized GM undergo dysregulation of cell death control and can present a cancer phenotype.

**Figure 2:**
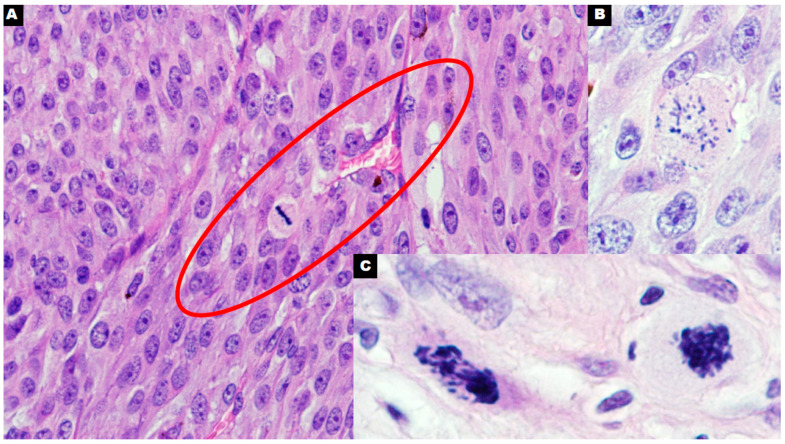
Melanoma with a single normal mitotic figure near small blood vessels with erythrocytes ((**A**), 400×) was observed. Pathologic mitoses with severe abnormal/asymmetrical morphology of chromatin in uterine leiomyosarcoma as a possible source of mitochondria during cytokinesis ((**B**,**C**); 600×).

The role of the GM in cancer cells remains unclear and should be investigated in future studies. We hypothesize that the mitochondrial transfer processes mentioned above could also involve the pathological GM. The GM can be transported in EVs to distant tissues, where it can serve as a signal to activate and proliferate dormant cells. The second possible hypothesis involves the interaction of the GM with immune cells and metastatic niche cells, which could be essential for preparing the TME for migrating cancer cells. Studies have not yet clarified the quantity and proportions of the GM compared with those of functional mitochondria in primary and metastatic cancer cells. Identifying markers for cell-free GM could help determine the number of circulating mitochondria and their potential role in assessing the risk of metastasis in patients with various types of cancer. Given the evidence suggesting the inefficiency of metastasis formation solely through circulating tumor cells [7,9], it is also important to explore the possibility that metastasis could spread via smaller entities, such as mitochondria. Perhaps cytokinesis in highly proliferative cancer cells (especially in tumors with an increased number of pathological mitoses) could also be a source of mitochondria released from dividing cells. The GM can enter previously healthy cells of distant organs and alter their metabolism and gene expression to produce a cancer phenotype (Figure 1 and Figure 2). Further studies are needed to investigate this hypothesis and identify the potential signaling pathways involved.

## 3. Vasculogenic Mimicry (VM)—Need for Nutrients or Method of Metastasis on the Basis of Interactions with Blood Cells?

Blood flow in cancer is crucial for supplying proliferating cancer cells with the necessary nutrients and oxygen. Angiogenesis is one of the best-examined processes for providing the blood supply to the TME. However, other mechanisms also exist, including vasculogenic mimicry (VM). VM was first described by Maniotis et al. and refers to a process by which cancer cells imitate endothelial cells to form the vasculature [47]. In VM, cancer cells can create vessel-like structures without the presence of endothelial cells [48]. Mechanisms underlying the VM formation are complex and associated with hypoxia. The decreased oxygen supply to the tumor leads to the activation of the hypoxia-inducible factor (HIF) pathway in cancer stem cells (CSCs) [49]. CSCs are a population of cancer cells that possess two features typical to stem cells: self-renewal and differentiation potential [50]. The activation of HIF-1 cascade in CSCs is responsible for the activation of pathways leading to epithelial-to-endothelial transition (EET) [49,51]. EET is a process of differentiation of CSCs into cells with endothelial characteristics [51]. The extracellular matrix remodeling and the EET are major processes leading to the formation of vessel-like structures in VM [49]. Two types of VM have been described: the tubular type and the patterned matrix type [52]. In the tubular type, cancer cells form vessel-like structures very similar to those of normal microvasculature [52,53]. The patterned matrix type is associated with the formation of morphologically dissimilar vessel-like structures surrounded by a matrix and clusters of cancer cells [52,53]. Regardless of the type, vessel-like canals originate from blood vessels and provide slow blood flow into cancer tissue, which enables cancer cells to interact with blood components [52]. The VM process is associated with the presence of PGCCs, which can create tubular vascular-like channels [54]. VM and the formation of PGCCs require similar hypoxia-induced pathways, and the stemness phenotype of PGCCs enables them to perform EET [54,55]. Studies of PGCCs have revealed that their stem-like features permit the cells to differentiate into erythrocyte-like cells which can enter the bloodstream [54]. VM has been observed in various cancer types, including melanoma, breast cancer, gastrointestinal cancers, and glioblastoma [47,53,56,57,58]. The presence of VM is associated with poor clinical outcomes and is characterized by increased invasion, metastasis, and resistance to antiangiogenic therapies [48,53]. In VM, cancer cells express VE-cadherin, which participates in the formation of vessel-like structures and activates pathways that promote survival and proliferation [53,59]. The structures formed during the VM process allow cancer cells to enter the bloodstream and interact with blood cells.

Interactions between different blood cells and cancer cells have important influences on CTC survival and the metastatic potential of tumors [60]. Studies have shown that cancer cells can interact with platelets to avoid the immune system and facilitate metastasis formation [61,62]. Once cancer cells enter the bloodstream, they can activate platelets by secreting different factors, including tissue factor and thrombin [63,64]. Activated platelets attach to CTCs and cover their surface, creating a protective layer and enabling cell interactions [60,63,65]. Platelets are able to both promote and inhibit cancer metastasis [63]. Prometastatic platelet function is associated with facilitating cancer cell immune escape, increasing adhesion ability and inhibiting cancer cell anoikis (a form of programmed cell death that occurs when cells detach from the surrounding extracellular matrix) [63]. Immune escape is connected mainly with the modification of antigens presented by cancer cells, which protects them from elimination by NK cells [63,66]. Platelets can also impair tumor progression and metastasis via modification of the cell cycle and apoptosis [63].

**Figure 3:**
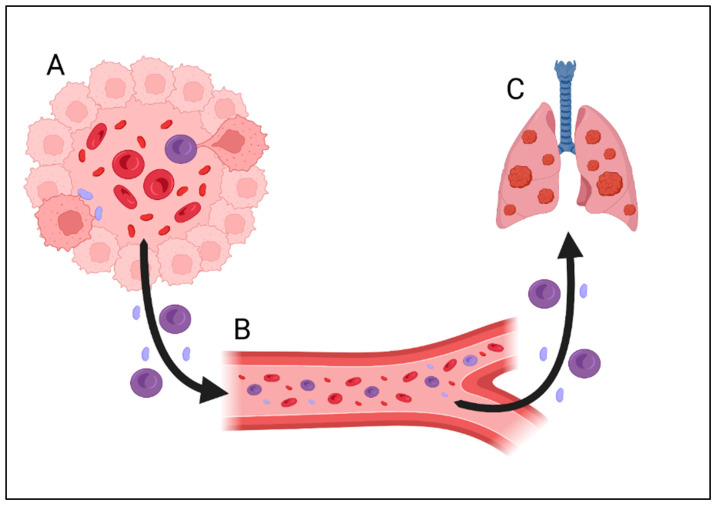
The proposed mechanism of cancer metastasis through vasculogenic mimicry. (**A**) “Education” of blood cells in vessel-like structures in cancer tissue. Cancer cells modify the function of blood cells and implant DNA into erythrocytes and platelets. (**B**) “Modified” platelets and erythrocytes are released into the circulation and migrate into the site of metastasis. (**C**) Metastasis formation occurs via the migration of “modified” cells as a result of interactions with niche cells, the activation of dormant cancer cells or the internalization of genetic material from cancer cells into healthy cells (acting as a “vector”).

**Figure 4:**
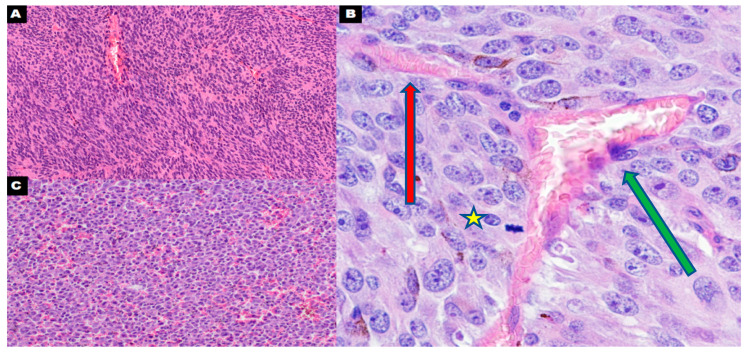
A regular blood vessel lined by endothelial cells in uveal melanoma ((**A**); 400×). Connection between the regular blood vessel lined by endothelial cells (green arrow) and the vascular channel partially lined by tumoral cells (red arrow) in uveal melanoma; asterisk: tumoral cell during mitosis in direct connection with the lumen of the vessel ((**B**); 600×). Dispersed vasculogenic mimicry (vascular channels lined only by tumor cells) in metastatic cutaneous melanoma ((**C**); 400×).

Cancer cells can also influence erythrocytes by altering their immunological function [67]. DNA fragments, likely originating from cancer cells, have been detected in mature red blood cells [68]. Tumor cells are able to produce vesicles containing RNA or DNA (both nuclear and mitochondrial) that can be transferred to other cells and translated into proteins, altering their function [69,70,71,72]. We postulate that VM and direct contact between tumor cells and the blood are involved in the metastasis process. Cancer cells possibly recruit platelets and erythrocytes to protect migrating cells from the immune system. Another potential mechanism involves these blood cells acting as vectors for cancer genetic material, transporting it to distant sites, such as bone marrow. This material could help prepare niches for future colonization by metastatic cancer cells, or it could be directly transferred to stem cells, which may then be altered to express cancer phenotypes. Studies on mouse models have not demonstrated the degree of deformability in cancer cells that is essential for their entry into the circulation. However, observations of human prostate cells suggest that circulating tumor cells (CTCs) exhibit a size and deformability similar to those of blood cells [73]. This finding raises the possibility that CTCs, which are responsible for metastasis, could actually be blood cells with cancer-like features. Moreover, studies that are used to diagnose the presence of CTCs in the blood often rely on the detection of surface antigens present in cancer cells and absent in healthy blood cells [73]. More studies, including studies on the greater quantity of antigens associated with the examination of cellular morphology, should be performed to determine the exact origin of CTCs. The potential role of stem cells in carcinogenesis has been previously proposed, with the idea that stem cells could differentiate into cancer cells [74]. Changes in the bone microenvironment in premetastatic disease, including changes in stem cell proportions, have been observed in previous studies, which suggests that cancer can modify the microenvironment before the development of metastasis [75] (Figure 3 and Figure 4). In our opinion, further research is needed to investigate whether the indirect evidence of the use of blood cells to disseminate and communicate with distant organs in cancer supports our hypothesis [76,77].

## 4. Polyploid Giant Cancer Cells—Dormant Locators or Migrating Pioneers

Polyploid giant cells (PGCs) are formed through the fusion of multiple mononuclear cells. The formation of PGCs is a common feature of the monocyte lineage and can occur in both physiological conditions (e.g., osteoclasts) and pathological conditions, such as Langhans giant cells in tuberculosis [78]. Polyploid giant cancer cells (PGCCs) are a subtype of PGC composed of fused cancer cells and are commonly observed in various cancer types, including melanoma, urothelial carcinoma, renal cell carcinoma, breast carcinoma, ovarian carcinoma, pancreatic adenocarcinoma, and prostate carcinoma [79]. PGCCs are a heterogenous group of cancer cells with diversified roles in cancer dormancy and metastasis, and their numbers are correlated with disease progression [80,81].

Different functions of PGCCs are associated with stemness features. Studies revealed that progeny cells of PGCCs possess stem cell markers including Oct-4, Sox-2, NANOG, CD44, and CD133 [54,82]. PGCCs exhibit characteristics similar to CSCs and have the ability to express CSC-related markers such as CD44 and CD133. Tumors derived from PGCCs express proteins associated with EMT, such as N-cadherin, Snail/Slug, and Twist. These proteins contribute to the increased metastatic and invasive capabilities of cancer cells [83].

The process of PGCC formation is initiated by cellular stress factors such as hypoxia, chemotherapy, radiotherapy, ROS production, viral infections, and other factors [79,84]. Hypoxia is the best known inductor and the hypoxia mimicking CoCl_2_ is commonly used for in vitro PGCC formation [85]. The exact mechanisms of PGCC induction are not fully understood; however, studies suggest similar pathways that are associated with VM [54,55]. Studies have revealed that important events leading to PGCC creation are connected with HIF-1 pathway activation and DNA damage [54,55,84].

PGCCs remain arrested in the G2/M phase of the cell cycle [81,86]. Cell cycle arrest is caused by impaired function of the cell cycle regulatory proteins CDC25B and CDC25C. During mitosis initiation, these proteins are normally translocated from the cytoplasm into the nucleus by cyclin B1. In dormant PGCCs, cyclin B1 is downregulated; however, it can also be highly expressed in PGCCs, which is associated with reactivation and production of daughter cells [81,85,86,87]. Studies of high-grade serous ovarian carcinoma revealed that PGCCs are able to undergo microevolution, which is responsible for resistance to chemotherapy and adaptation to metastasis [88,89]. During their dormant phase, PGCCs undergo endomitotic nuclear divisions without cytokinesis, increasing the quantity of genetic material [88]. Some factors, e.g., the cellular stress caused by radiotherapy or chemotherapy, can activate dormant PGCCs [90,91,92,93]. The activated PGCCs divide by neosis, which consists of amitotic nuclear fragmentation and cytokinesis, to produce blastomere-like organoids filled with daughter cells [90,92]. Moreover the stemness of PGCCs enables them to differentiate into different cellular lines, including cells of each germ layer, as a response to signalizing factors in the TME [54,55,82]. Fecundity daughter cells can be released into the TME, where they undergo mitotic divisions, resulting in tumor formation [88,92,94]. While PGCCs are widely described as dormant cells formed by fused cancer cells [64,76], there is also a population of PGCCs that express surface proteins typical of immune cells (e.g., macrophages), allowing them to migrate through blood vessels and contribute to metastasis [95].

**Figure 5:**
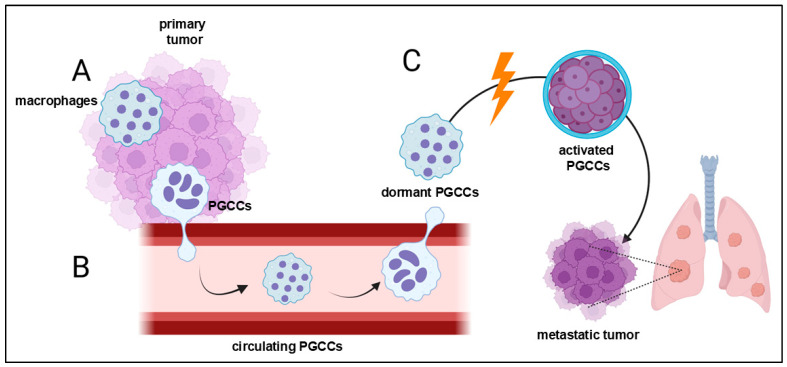
Hypothetical role of polyploid giant cancer cells (PGCCs) in metastasis. (**A**) PGCCs are generated in primary tumors by the fusion of cancer cells or by the fusion of cancer cells with macrophages. (**B**) PGCCs, which are hybrids of macrophages and cancer cells, can enter the circulation and migrate to sites of metastasis. (**C**) PGCCs residing in metastatic niches are activated from a dormant state. Activated PGCCs undergo neosis and release daughter cells, which create metastatic tumors.

**Figure 6:**
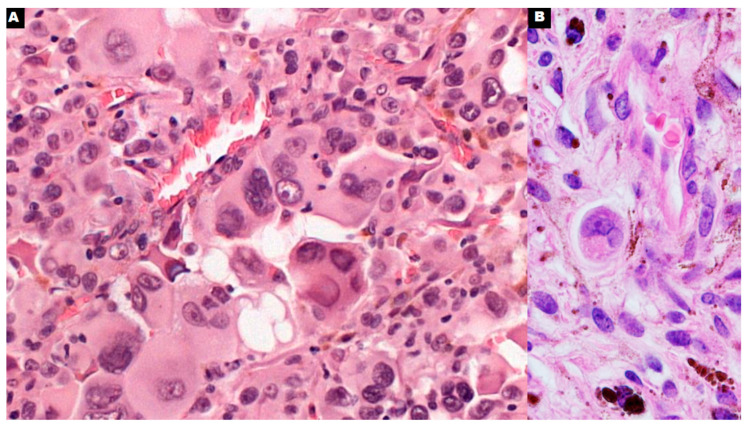
Polyploid giant cancer cells of uveal melanoma in direct contact with small blood vessels ((**A**,**B**); 600×).

**Figure 7:**
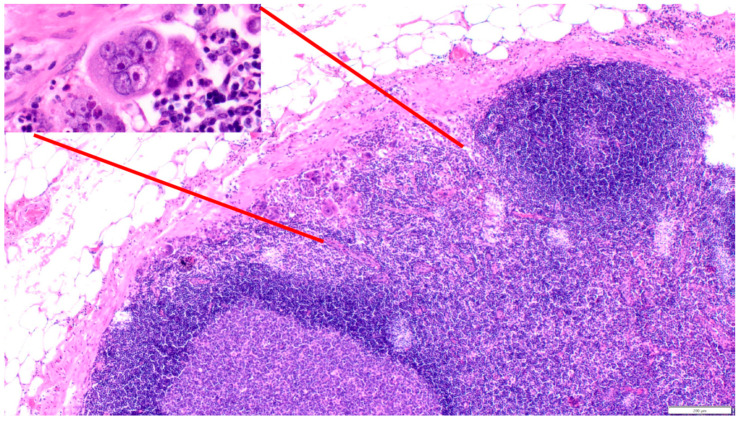
Polyploid giant cancer cells in chemoresistant nodal metastases of poorly differentiated gastric adenocarcinoma (200×); inset: multinucleated giant cell with several highly atypical nuclei (600×).

Macrophages constitute another significant cell population associated with cancer metastasis and the possible formation of PGCCs [96]. As innate immune cells with phagocytic capabilities, macrophages play dual roles in cancer, contributing to both antitumor immunity and disease progression. The role of macrophages in cancer is diverse; for that reason, three novel types of macrophages have been identified [97]. The first type is regular tumor-associated macrophages (TAMs), which are important elements of the TME. The second type is the population of giant cells that contain cancer proteins and DNA fragments but also express the macrophage-specific protein CD14; these cells are referred to as cancer-associated macrophage-like cells (CAMLs) [97]. CAMLs carry the DNA and proteome from phagocytosed cancer cells and can migrate into blood vessels to perform immune functions. The third type of cancer-associated macrophage is giant hybrid cells (GHCs), which are formed through the fusion of macrophages and cancer cells and contain functional cancer cell nuclei. GHCs leave the TME in large numbers and enter the circulation as circulating hybrid cells (CHCs) [95] (Figure 5, Figure 6 and Figure 7). The immunotolerance provided by macrophage surface markers and the ability to promote neovascularization through the expression of TIE-2/CD146 support the hypothesis that PGCCs with a macrophage phenotype could serve as an effective mechanism for cancer dissemination [95].

## 5. Discussion

Ghost mitochondria (GM), vasculogenic mimicry (VM), and polyploid giant cancer cells (PGCCs) are three underestimated concepts that may help elucidate the process of cancer metastasis. Currently, there are two active clinical trials on VM (NCT03600233) and PGCCs (NCT06389201), discussed hereunder. We also discuss the correlation between PGCCs and VM formation in several neoplasms.

Specific dormant PGCCs have garnered significant attention due to their correlation with the clinical risk of recurrence in nasopharyngeal carcinoma (NPC). In a study by You et al., the authors demonstrate that autophagy serves as a crucial mechanism for the induction of PGCCs. Furthermore, the pharmacological inhibition of autophagy markedly reduced the formation of PGCCs, leading to a significant decrease in metastasis and an improvement in survival rates in a mouse model [98]. Consequently, one clinical trial (NCT06389201) will evaluate whether an autophagy inhibitor, hydroxychloroquine (HCQ), inhibits the development of therapy-induced dormant PGCCs. This approach has the potential to reduce the risk of recurrence and metastasis in patients with NPC.

CVM-1118 (foslinanib, TRX-818) is a novel small molecule with a high potential for inhibiting VM formation via targeting TNF receptor-associated protein 1 (TRAP1) [99]. The notable occurrence of VM has been documented in neuroendocrine tumors (NETs). Furthermore, VM has been identified as a component of various microvascular changes in mouse models of pancreatic NETs. This compound has demonstrated significant anticancer activity across various human cancer cell lines. The safety profile of CVM-1118 in human subjects is currently being assessed through a phase 1 clinical study. The primary objective of the phase 2 study (NCT03600233) is to further evaluate the efficacy of CVM-1118 in patients diagnosed with advanced NETs [100].

The number of PGCCs is correlated with the formation of VM human glioma. The walls of VM exhibit positive (or negative) staining for PAS and are negative for CD31 staining. Furthermore, the prevalence of VM is significantly greater in high-grade gliomas compared to low-grade gliomas [101]. Similarly, the number of PGCCs is associated with VM formation in human ovarian cancer. In a study by Zhang et al., the metastatic foci of ovarian carcinoma exhibited the highest prevalence of PGCCs and VM. An increase in the number of PGCCs and VM was observed in correlation with the grade of ovarian carcinoma. PGCCs were found to generate erythroid cells through a budding process, and collectively, these cells contributed to the formation of VM [102]. The quantity of PGCCs and VM was also correlated with the differentiation of colorectal cancer. Additionally, daughter cells that originate from PGCCs may facilitate lymph node metastasis through the expression of EMT-related proteins. PGCCs, along with newly generated erythroid cells, contribute to the formation of VM structures in colorectal cancer [103]. In another study, Liu et al. found that primary anorectal malignant melanomas exhibiting lymph node metastasis demonstrate a higher incidence of PGCCs and VM compared to those without metastasis. Authors confirmed that PGCCs and their generated erythroid cells have the ability to form VM [104]. Thus, the number of PGCCs is correlated with disease development, progression, and formation of VM in glioma, ovarian carcinoma, colorectal cancer, and malignant melanoma.

Blocking mitochondrial metabolism in cancer is a relatively new concept that led to clinical trials targeting the mitochondrial energy homeostasis essential for cancer proliferation [105,106]. The new emerging field of cancer therapy could be identifying signaling pathways associated with GM. The GCN2/Akt kinase signaling cascade seems to be one of the most important targets in cancers with GM [43]. Blocking that pathway could be a major component of metastatic cancer therapy that might possibly result in deceleration of tumor progression and extend survival in patients with advanced disease [43]. In vitro studies of available GCN2 inhibitors revealed promising effects on inducing cell death in chronic diseases; however, its value in cancer therapy could be more complex and should be determined in further clinical trials [107]. VM seems to be a promising target in therapy of advanced cancers. Studies of fasudil revealed that the drug inhibited vascular-like structure formation and tumor growth in melanoma mice xenografts [76]. Studies of in vitro glioma models showed that histone deacetylase inhibitors inhibit the VM and could potentially be used in therapy [77]. Achieving a better understanding of VM and determining the effects of blocking VM pathways on overall patient outcome are important directions for further studies. PGCCs are associated with drug resistance in many cancer types. The utilization of drugs targeting PGCC endoreplication provides a possible additional therapy, eliminating drug resistance. Mifepristone promotes apoptosis of endoreplicating PGCCs and blocks their formation. The use of mifepristone with olaparib was more effective in restricting tumor growth than each of the drugs in a monotherapy ovarian cancer model [108]. Targeting GM, VM, and PGCCs is an emerging field for developing new cancer therapies in advanced metastatic cancers.

## 6. Conclusions

In summary, this article highlights the multifaceted nature of metastatic processes and emphasizes the necessity of incorporating a wide range of scientific viewpoints to enhance our comprehension. Targeting GM, VM, and PGCCs is an emerging field for developing new cancer therapies in advanced metastatic cancers. The future of metastasis research is anchored in the ongoing investigation of these emerging paradigms and their application in clinical settings. These concepts may lead to the development of innovative therapeutic strategies.

## References

[1] Dillekås H., Rogers M.S., Straume O. (2019). Are 90% of Deaths from Cancer Caused by Metastases?. Cancer Med..

[2] Siegel R.L., Miller K.D., Wagle N.S., Jemal A. (2023). Cancer Statistics, 2023. CA Cancer J. Clin..

[3] Gennari A., Conte P., Rosso R., Orlandini C., Bruzzi P. (2005). Survival of Metastatic Breast Carcinoma Patients over a 20-year Period. Cancer.

[4] Mani K., Deng D., Lin C., Wang M., Hsu M.L., Zaorsky N.G. (2024). Causes of Death among People Living with Metastatic Cancer. Nat. Commun..

[5] Paget S. (1889). The distribution of secondary growths in cancer of the breast. Lancet.

[6] Lin D., Shen L., Luo M., Zhang K., Li J., Yang Q., Zhu F., Zhou D., Zheng S., Chen Y. (2021). Circulating Tumor Cells: Biology and Clinical Significance. Signal Transduct. Target. Ther..

[7] Massagué J., Obenauf A.C. (2016). Metastatic Colonization by Circulating Tumour Cells. Nature.

[8] Fares J., Fares M.Y., Khachfe H.H., Salhab H.A., Fares Y. (2020). Molecular Principles of Metastasis: A Hallmark of Cancer Revisited. Signal Transduct. Target. Ther..

[9] Luzzi K.J., MacDonald I.C., Schmidt E.E., Kerkvliet N., Morris V.L., Chambers A.F., Groom A.C. (1998). Multistep Nature of Metastatic Inefficiency: Dormancy of Solitary Cells after Successful Extravasation and Limited Survival of Early Micrometastases. Am. J. Pathol..

[10] Martin O.A., Anderson R.L., Narayan K., MacManus M.P. (2017). Does the Mobilization of Circulating Tumour Cells during Cancer Therapy Cause Metastasis?. Nat. Rev. Clin. Oncol..

[11] Su J.-X., Li S.-J., Zhou X.-F., Zhang Z.-J., Yan Y., Liu S.-L., Qi Q. (2023). Chemotherapy-Induced Metastasis: Molecular Mechanisms and Clinical Therapies. Acta Pharmacol. Sin..

[12] Warburg O. (1925). The Metabolism of Carcinoma Cells. J. Cancer Res..

[13] Seyfried T.N. (2015). Cancer as a Mitochondrial Metabolic Disease. Front. Cell Dev. Biol..

[14] Kaipparettu B.A., Ma Y., Park J.H., Lee T.L., Zhang Y., Yotnda P., Creighton C.J., Chan W.Y., Wong L.J.C. (2013). Crosstalk from Non-Cancerous Mitochondria Can Inhibit Tumor Properties of Metastatic Cells by Suppressing Oncogenic Pathways. PLoS ONE.

[15] Elliott R.L., Jiang X.P., Head J.F. (2012). Mitochondria Organelle Transplantation: Introduction of Normal Epithelial Mitochondria into Human Cancer Cells Inhibits Proliferation and Increases Drug Sensitivity. Breast Cancer Res. Treat..

[16] Shay J.W. (1988). Cytoplasmic Suppression of Tumorigenicity in Reconstructed Mouse Cells. Cancer Res..

[17] Mckinnell R.G., Deggins B.A., Labat D.D. (1969). Transplantation of Pluripotential Nuclei from Triploid Frog Tumors. Science.

[18] Ishikawa K., Takenaga K., Akimoto M., Koshikawa N., Yamaguchi A., Imanishi H., Nakada K., Honma Y., Hayashi J.I. (2008). ROS-Generating Mitochondrial DNA Mutations Can Regulate Tumor Cell Metastasis. Science.

[19] Garimella S.V., Gampa S.C., Chaturvedi P. (2023). Mitochondria in Cancer Stem Cells: From an Innocent Bystander to a Central Player in Therapy Resistance. Stem Cells Cloning.

[20] Cheng X., Henick B.S., Cheng K. (2024). Anticancer Therapy Targeting Cancer-Derived Extracellular Vesicles. ACS Nano.

[21] Ippolito L., Morandi A., Taddei M.L., Parri M., Comito G., Iscaro A., Raspollini M.R., Magherini F., Rapizzi E., Masquelier J. (2019). Cancer-Associated Fibroblasts Promote Prostate Cancer Malignancy via Metabolic Rewiring and Mitochondrial Transfer. Oncogene.

[22] Zhang Z.G., Buller B., Chopp M. (2019). Exosomes—Beyond Stem Cells for Restorative Therapy in Stroke and Neurological Injury. Nat. Rev. Neurol..

[23] Choi D., Montermini L., Jeong H., Sharma S., Meehan B., Rak J. (2019). Mapping Subpopulations of Cancer Cell-Derived Extracellular Vesicles and Particles by Nano-Flow Cytometry. ACS Nano.

[24] Nawaz M., Camussi G., Valadi H., Nazarenko I., Ekström K., Wang X., Principe S., Shah N., Ashraf N.M., Fatima F. (2014). The Emerging Role of Extracellular Vesicles as Biomarkers for Urogenital Cancers. Nat. Rev. Urol..

[25] Stier A. (2021). Human Blood Contains Circulating Cell-Free Mitochondria, but Are They Really Functional?. Am. J. Physiol. Endocrinol. Metab..

[26] Stephens O.R., Grant D., Frimel M., Wanner N., Yin M., Willard B., Erzurum S.C., Asosingh K. (2020). Characterization and Origins of Cell-Free Mitochondria in Healthy Murine and Human Blood. Mitochondrion.

[27] Mohd Khair S.Z.N., Abd Radzak S.M., Mohamed Yusoff A.A. (2021). The Uprising of Mitochondrial DNA Biomarker in Cancer. Dis. Markers.

[28] Jang S.C., Crescitelli R., Cvjetkovic A., Belgrano V., Olofsson Bagge R., Sundfeldt K., Ochiya T., Kalluri R., Lötvall J. (2019). Mitochondrial Protein Enriched Extracellular Vesicles Discovered in Human Melanoma Tissues Can Be Detected in Patient Plasma. J. Extracell. Vesicles.

[29] Boudreau L.H., Duchez A.C., Cloutier N., Soulet D., Martin N., Bollinger J., Paré A., Rousseau M., Naika G.S., Lévesque T. (2014). Platelets Release Mitochondria Serving as Substrate for Bactericidal Group IIA-Secreted Phospholipase a to Promote Inflammation. Blood.

[30] Lin L., Xu H., Bishawi M., Feng F.F., Samy K., Truskey G., Barbas A.S., Kirk A.D., Brennan T.V. (2019). Circulating Mitochondria in Organ Donors Promote Allograft Rejection. Am. J. Transplant..

[31] Sahinbegovic H., Jelinek T., Hrdinka M., Bago J.R., Turi M., Sevcikova T., Kurtovic-Kozaric A., Hajek R., Simicek M. (2020). Intercellular Mitochondrial Transfer in the Tumor Microenvironment. Cancers.

[32] Chang J.C., Chang H.S., Wu Y.C., Cheng W.L., Lin T.T., Chang H.J., Kuo S.J., Chen S.T., Liu C.S. (2019). Mitochondrial Transplantation Regulates Antitumour Activity, Chemoresistance and Mitochondrial Dynamics in Breast Cancer. J. Exp. Clin. Cancer Res..

[33] Zampieri L.X., Silva-Almeida C., Rondeau J.D., Sonveaux P. (2021). Mitochondrial Transfer in Cancer: A Comprehensive Review. Int. J. Mol. Sci..

[34] Guo X., Can C., Liu W., Wei Y., Yang X., Liu J., Jia H., Jia W., Wu H., Ma D. (2023). Mitochondrial Transfer in Hematological Malignancies. Biomark. Res..

[35] Hayes J.D., Dinkova-Kostova A.T., Tew K.D. (2020). Oxidative Stress in Cancer. Cancer Cell.

[36] Sellers K., Fox M.P., Ii M.B., Slone S.P., Higashi R.M., Miller D.M., Wang Y., Yan J., Yuneva M.O., Deshpande R. (2015). Pyruvate Carboxylase Is Critical for Non-Small-Cell Lung Cancer Proliferation. J. Clin. Investig..

[37] Anderson R.G., Ghiraldeli L.P., Pardee T.S. (2018). Mitochondria in Cancer Metabolism, an Organelle Whose Time Has Come?. Biochim. Biophys. Acta Rev. Cancer.

[38] Burt R., Dey A., Aref S., Aguiar M., Akarca A., Bailey K., Day W., Hooper S., Kirkwood A., Kirschner K. (2019). Activated Stromal Cells Transfer Mitochondria to Rescue Acute Lymphoblastic Leukemia Cells from Oxidative Stress. Blood.

[39] Moschoi R., Imbert V., Nebout M., Chiche J., Mary D., Prebet T., Saland E., Castellano R., Pouyet L., Collette Y. (2016). Protective Mitochondrial Transfer from Bone Marrow Stromal Cells to Acute Myeloid Leukemic Cells during Chemotherapy. Blood.

[40] Saha T., Dash C., Jayabalan R., Khiste S., Kulkarni A., Kurmi K., Mondal J., Majumder P.K., Bardia A., Jang H.L. (2022). Intercellular Nanotubes Mediate Mitochondrial Trafficking between Cancer and Immune Cells. Nat. Nanotechnol..

[41] Levoux J., Prola A., Lafuste P., Gervais M., Chevallier N., Koumaiha Z., Kefi K., Braud L., Schmitt A., Yacia A. (2021). Platelets Facilitate the Wound-Healing Capability of Mesenchymal Stem Cells by Mitochondrial Transfer and Metabolic Reprogramming. Cell Metab..

[42] Zhang W., Zhou H., Li H., Mou H., Yinwang E., Xue Y., Wang S., Zhang Y., Wang Z., Chen T. (2023). Cancer Cells Reprogram to Metastatic State through the Acquisition of Platelet Mitochondria. Cell Rep..

[43] Ghosh J.C., Perego M., Agarwal E., Bertolini I., Wang Y., Goldman A.R., Tang H.Y., Kossenkov A.V., Libby C.J., Languino L.R. (2022). Ghost Mitochondria Drive Metastasis through Adaptive GCN2/Akt Therapeutic Vulnerability. Proc. Natl. Acad. Sci. USA.

[44] Altieri D.C. (2023). Mitochondria in Cancer: Clean Windmills or Stressed Tinkerers?. Trends Cell Biol..

[45] Li H., Ruan Y., Zhang K., Jian F., Hu C., Miao L., Gong L., Sun L., Zhang X., Chen S. (2016). Mic60/Mitofilin Determines MICOS Assembly Essential for Mitochondrial Dynamics and MtDNA Nucleoid Organization. Cell Death Differ..

[46] Kossenkov A.V., Milcarek A., Notta F., Jang G.H., Wilson J.M., Gallinger S., Zhou D.C., Ding L., Ghosh J.C., Perego M. (2022). Mitochondrial Fitness and Cancer Risk. PLoS ONE.

[47] Maniotis A.J., Folberg R., Hess A., Seftor E.A., Gardner L.M.G., Pe’er J., Trent J.M., Meltzer P.S., Hendrix M.J.C. (1999). Vascular Channel Formation by Human Melanoma Cells in Vivo and in Vitro: Vasculogenic Mimicry. Am. J. Pathol..

[48] Luo Q., Wang J., Zhao W., Peng Z., Liu X., Li B., Zhang H., Shan B., Zhang C., Duan C. (2020). Vasculogenic Mimicry in Carcinogenesis and Clinical Applications. J. Hematol. Oncol..

[49] Wei X., Chen Y., Jiang X., Peng M., Liu Y., Mo Y., Ren D., Hua Y., Yu B., Zhou Y. (2021). Mechanisms of Vasculogenic Mimicry in Hypoxic Tumor Microenvironments. Mol. Cancer.

[50] Bisht S., Nigam M., Kunjwal S.S., Sergey P., Mishra A.P., Sharifi-Rad J. (2022). Cancer Stem Cells: From an Insight into the Basics to Recent Advances and Therapeutic Targeting. Stem Cells Int..

[51] Sun B., Zhang D., Zhao N., Zhao X. (2017). Epithelial-to-Endothelial Transition and Cancer Stem Cells: Two Cornerstones of Vasculogenic Mimicry in Malignant Tumors. Oncotarget.

[52] Lapkina E.Z., Esimbekova A.R., Ruksha T.G. (2023). Vasculogenic Mimicry. Arkh Patol..

[53] Andonegui-Elguera M.A., Alfaro-Mora Y., Cáceres-Gutiérrez R., Caro-Sánchez C.H.S., Herrera L.A., Díaz-Chávez J. (2020). An Overview of Vasculogenic Mimicry in Breast Cancer. Front. Oncol..

[54] Saini G., Joshi S., Garlapati C., Li H., Kong J., Krishnamurthy J., Reid M.D., Aneja R. (2022). Polyploid Giant Cancer Cell Characterization: New Frontiers in Predicting Response to Chemotherapy in Breast Cancer. Semin. Cancer Biol..

[55] Liu P., Wang L., Yu H. (2024). Polyploid Giant Cancer Cells: Origin, Possible Pathways of Formation, Characteristics, and Mechanisms of Regulation. Front. Cell Dev. Biol..

[56] Liu Y., Sun B., Lin Y., Deng H., Wang X., Xu C., Wang K., Yu N., Liu R., Han M. (2024). Lysyl Oxidase Promotes the Formation of Vasculogenic Mimicry in Gastric Cancer through PDGF-PDGFR Pathway. J. Cancer.

[57] Han C., Sun B., Zhao X., Zhang Y., Gu Q., Liu F., Zhao N., Wu L. (2017). Phosphorylation of STAT3 Promotes Vasculogenic Mimicry by Inducing Epithelial-to-Mesenchymal Transition in Colorectal Cancer. Technol. Cancer Res. Treat..

[58] Lizárraga-Verdugo E., Avendaño-Félix M., Bermúdez M., Ramos-Payán R., Pérez-Plasencia C., Aguilar-Medina M. (2020). Cancer Stem Cells and Its Role in Angiogenesis and Vasculogenic Mimicry in Gastrointestinal Cancers. Front. Oncol..

[59] Paulis Y.W.J., Soetekouw P.M.M.B., Verheul H.M.W., Tjan-Heijnen V.C.G., Griffioen A.W. (2010). Signalling Pathways in Vasculogenic Mimicry. Biochim. Biophys. Acta Rev. Cancer.

[60] Pereira-Veiga T., Schneegans S., Pantel K., Wikman H. (2022). Circulating Tumor Cell-Blood Cell Crosstalk: Biology and Clinical Relevance. Cell Rep..

[61] Patel D., Thankachan S., Sreeram S., Kavitha K.P., Suresh P.S. (2023). The Role of Tumor-Educated Platelets in Ovarian Cancer: A Comprehensive Review and Update. Pathol. Res. Pract..

[62] Best M.G., Sol N., In ‘t Veld S.G.J.G., Vancura A., Muller M., Niemeijer A.L.N., Fejes A.V., Tjon Kon Fat L.A., Huis In ‘t Veld A.E., Leurs C. (2017). Swarm Intelligence-Enhanced Detection of Non-Small-Cell Lung Cancer Using Tumor-Educated Platelets. Cancer Cell.

[63] Bian X., Yin S., Yang S., Jiang X., Wang J., Zhang M., Zhang L. (2022). Roles of Platelets in Tumor Invasion and Metastasis: A Review. Heliyon.

[64] Ruf W., Yokota N., Schaffner F. (2010). Tissue Factor in Cancer Progression and Angiogenesis. Thromb. Res..

[65] Li S., Lu Z., Wu S., Chu T., Li B., Qi F., Zhao Y., Nie G. (2024). The Dynamic Role of Platelets in Cancer Progression and Their Therapeutic Implications. Nat. Rev. Cancer.

[66] Cluxton C.D., Spillane C., O’Toole S.A., Sheils O., Gardiner C.M., O’Leary J.J. (2019). Suppression of Natural Killer Cell NKG2D and CD226 Anti-Tumour Cascades by Platelet Cloaked Cancer Cells: Implications for the Metastatic Cascade. PLoS ONE.

[67] Karsten E., Breen E., McCracken S.A., Clarke S., Herbert B.R. (2020). Red Blood Cells Exposed to Cancer Cells in Culture Have Altered Cytokine Profiles and Immune Function. Sci. Rep..

[68] Liang N., Jiao Z., Zhang C., Wu Y., Wang T., Li S., Wang Y., Song T., Chen J.Q., Liang H. (2023). Mature Red Blood Cells Contain Long DNA Fragments and Could Acquire DNA from Lung Cancer Tissue. Adv. Sci..

[69] Nilsson R.J.A., Balaj L., Hulleman E., Van Rijn S., Pegtel D.M., Walraven M., Widmark A., Gerritsen W.R., Verheul H.M., Vandertop W.P. (2011). Blood Platelets Contain Tumor-Derived RNA Biomarkers. Blood.

[70] Girardot M., Pecquet C., Boukour S., Knoops L., Ferrant A., Vainchenker W., Giraudier S., Constantinescu S.N. (2010). MiR-28 Is a Thrombopoietin Receptor Targeting MicroRNA Detected in a Fraction of Myeloproliferative Neoplasm Patient Platelets. Blood.

[71] Nilsson J., Skog J., Nordstrand A., Baranov V., Mincheva-Nilsson L., Breakefield X.O., Widmark A. (2009). Prostate Cancer-Derived Urine Exosomes: A Novel Approach to Biomarkers for Prostate Cancer. Br. J. Cancer.

[72] Ma J., Zhang H., Tang K., Huang B. (2020). Tumor-Derived Microparticles in Tumor Immunology and Immunotherapy. Eur. J. Immunol..

[73] Bagnall J.S., Byun S., Begum S., Miyamoto D.T., Hecht V.C., Maheswaran S., Stott S.L., Toner M., Hynes R.O., Manalis S.R. (2015). Deformability of Tumor Cells versus Blood Cells. Sci. Rep..

[74] Afify S.M., Seno M. (2019). Conversion of Stem Cells to Cancer Stem Cells: Undercurrent of Cancer Initiation. Cancers.

[75] Foster B.M., Shi L., Harris K.S., Patel C., Surratt V.E., Langsten K.L., Kerr B.A. (2022). Bone Marrow-Derived Stem Cell Factor Regulates Prostate Cancer-Induced Shifts in Pre-Metastatic Niche Composition. Front. Oncol..

[76] Xia Y., Cai X.Y., Fan J.Q., Zhang L.L., Ren J.H., Chen J., Li Z.Y., Zhang R.G., Zhu F., Wu G. (2015). Rho Kinase Inhibitor Fasudil Suppresses the Vasculogenic Mimicry of B16 Mouse Melanoma Cells Both in Vitro and in Vivo. Mol. Cancer Ther..

[77] Colucci-D’amato L., Pastorino O., Teresa Gentile M., Mancini A., Del Gaudio N., Di Costanzo A., Bajetto A., Franco P., Altucci L., Florio T. (2019). Histone Deacetylase Inhibitors Impair Vasculogenic Mimicry from Glioblastoma Cells. Cancers.

[78] Hazra S., Kalyan Dinda S., Kumar Mondal N., Hossain S.R., Datta P., Yasmin Mondal A., Malakar P., Manna D. (2023). Giant Cells: Multiple Cells Unite to Survive. Front. Cell Infect. Microbiol..

[79] Zhou X., Zhou M., Zheng M., Tian S., Yang X., Ning Y., Li Y., Zhang S. (2022). Polyploid Giant Cancer Cells and Cancer Progression. Front. Cell Dev. Biol..

[80] Jiao Y., Yu Y., Zheng M., Yan M., Wang J., Zhang Y., Zhang S. (2024). Dormant Cancer Cells and Polyploid Giant Cancer Cells: The Roots of Cancer Recurrence and Metastasis. Clin. Transl. Med..

[81] Liu K., Zheng M., Zhao Q., Zhang K., Li Z., Fu F., Zhang H., Du J., Li Y., Zhang S. (2020). Different P53 Genotypes Regulating Different Phosphorylation Sites and Subcellular Location of CDC25C Associated with the Formation of Polyploid Giant Cancer Cells. J. Exp. Clin. Cancer Res..

[82] Zhang S., Mercado-Uribe I., Xing Z., Sun B., Kuang J., Liu J. (2014). Generation of Cancer Stem-like Cells through the Formation of Polyploid Giant Cancer Cells. Oncogene.

[83] Wang X., Zheng M., Fei F., Li C., Du J., Liu K., Li Y., Zhang S. (2019). EMT-Related Protein Expression in Polyploid Giant Cancer Cells and Their Daughter Cells with Different Passages after Triptolide Treatment. Med. Oncol..

[84] Liu Y., Shi Y., Wu M., Liu J., Wu H., Xu C., Chen L. (2022). Hypoxia-Induced Polypoid Giant Cancer Cells in Glioma Promote the Transformation of Tumor-Associated Macrophages to a Tumor-Supportive Phenotype. CNS Neurosci. Ther..

[85] Lopez-Sánchez L.M., Jimenez C., Valverde A., Hernandez V., Peñarando J., Martinez A., Lopez-Pedrera C., Muñoz-Castañeda J.R., De La Haba-Rodríguez J.R., Aranda E. (2014). CoCl_2_, a Mimic of Hypoxia, Induces Formation of Polyploid Giant Cells with Stem Characteristics in Colon Cancer. PLoS ONE.

[86] Fei F., Qu J., Liu K., Li C., Wang X., Li Y., Zhang S. (2019). The Subcellular Location of Cyclin B1 and CDC25 Associated with the Formation of Polyploid Giant Cancer Cells and Their Clinicopathological Significance. Lab. Investig..

[87] Lu P., White-Gilbertson S., Beeson G., Beeson C., Ogretmen B., Norris J., Voelkel-Johnson C. (2021). Ceramide Synthase 6 Maximizes P53 Function to Prevent Progeny Formation from Polyploid Giant Cancer Cells. Cancers.

[88] Li X., Zhong Y., Zhang X., Sood A.K., Liu J. (2023). Spatiotemporal View of Malignant Histogenesis and Macroevolution via Formation of Polyploid Giant Cancer Cells. Oncogene.

[89] Zhao S., Xing S., Wang L., Ouyang M., Liu S., Sun L., Yu H. (2023). IL-1β Is Involved in Docetaxel Chemoresistance by Regulating the Formation of Polyploid Giant Cancer Cells in Non-Small Cell Lung Cancer. Sci. Rep..

[90] Niu N., Mercado-Uribe I., Liu J. (2017). Dedifferentiation into Blastomere-like Cancer Stem Cells via Formation of Polyploid Giant Cancer Cells. Oncogene.

[91] Erenpreisa J.A., Cragg M.S., Fringes B., Sharakhov I., Illidge T.M. (2000). Release of Mitotic Descendants by Giant Cells from Irradiated Burkitt’s Lymphoma Cell Lines. Cell Biol. Int..

[92] Sundaram M., Guernsey D.L., Rajaraman M.M., Rajaraman R. (2004). Neosis: A Novel Type of Cell Division in Cancer. Cancer Biol. Ther..

[93] Zhang Z., Feng X., Deng Z., Cheng J., Wang Y., Zhao M., Zhao Y., He S., Huang Q. (2021). Irradiation-Induced Polyploid Giant Cancer Cells Are Involved in Tumor Cell Repopulation via Neosis. Mol. Oncol..

[94] Liu J. (2020). The “Life Code”: A Theory That Unifies the Human Life Cycle and the Origin of Human Tumors. Semin. Cancer Biol..

[95] Adams D.L., Martin S.S., Alpaugh R.K., Charpentier M., Tsai S., Bergan R.C., Ogden I.M., Catalona W., Chumsri S., Tang C.M. (2014). Circulating Giant Macrophages as a Potential Biomarker of Solid Tumors. Proc. Natl. Acad. Sci. USA.

[96] Mantovani A., Allavena P., Marchesi F., Garlanda C. (2022). Macrophages as Tools and Targets in Cancer Therapy. Nat. Rev. Drug Discov..

[97] Sutton T.L., Patel R.K., Anderson A.N., Bowden S.G., Whalen R., Giske N.R., Wong M.H. (2022). Circulating Cells with Macrophage-like Characteristics in Cancer: The Importance of Circulating Neoplastic-Immune Hybrid Cells in Cancer. Cancers.

[98] You B., Xia T., Gu M., Zhang Z., Zhang Q., Shen J., Fan Y., Yao H., Pan S., Lu Y. (2022). AMPK-MTOR-Mediated Activation of Autophagy Promotes Formation of Dormant Polyploid Giant Cancer Cells. Cancer Res..

[99] Shen L., Chen Y.L., Huang C.C., Shyu Y.C., Seftor R.E.B., Seftor E.A., Hendrix M.J.C., Chien D.S., Chu Y.W. (2023). CVM-1118 (Foslinanib), a 2-Phenyl-4-Quinolone Derivative, Promotes Apoptosis and Inhibits Vasculogenic Mimicry via Targeting TRAP1. Pathol. Oncol. Res..

[100] Su W.-C., Chen M.H., Bai L.-Y., Chen J.-S., Chen Y.-Y., Shih Y.-H., Wu I.-C., Gutheil J., Melink T.J., Chu Y.-W. (2023). CVM-1118: A Potent Oral Anti-Vasculogenic Mimicry (VM) Agent in Patients with Advanced Neuroendocrine Tumors (NETs)-A Phase IIa Study. Am. Soc. Clin. Oncol..

[101] Qu Y., Zhang L., Rong Z., He T., Zhang S. (2013). Number of Glioma Polyploid Giant Cancer Cells (PGCCs) Associated with Vasculogenic Mimicry Formation and Tumor Grade in Human Glioma. J. Exp. Clin. Cancer Res..

[102] Zhang L., Ding P., Lv H., Zhang D., Liu G., Yang Z., Li Y., Liu J., Zhang S. (2014). Number of Polyploid Giant Cancer Cells and Expression of EZH2 Are Associated with VM Formation and Tumor Grade in Human Ovarian Tumor. Biomed. Res. Int..

[103] Zhang D., Yang X., Yang Z., Fei F., Li S., Qu J., Zhang M., Li Y., Zhang X., Zhang S. (2017). Daughter Cells and Erythroid Cells Budding from PGCCs and Their Clinicopathological Significances in Colorectal Cancer. J. Cancer.

[104] Liu G., Wang Y., Fei F., Wang X., Li C., Liu K., Du J., Cao Y., Zhang S. (2018). Clinical Characteristics and Preliminary Morphological Observation of 47 Cases of Primary Anorectal Malignant Melanomas. Melanoma Res..

[105] Sainero-Alcolado L., Liaño-Pons J., Ruiz-Pérez M.V., Arsenian-Henriksson M. (2022). Targeting Mitochondrial Metabolism for Precision Medicine in Cancer. Cell Death Differ..

[106] Vasan K., Werner M., Chandel N.S. (2020). Mitochondrial Metabolism as a Target for Cancer Therapy. Cell Metab..

[107] Piecyk M., Ferraro-Peyret C., Laville D., Perros F., Chaveroux C. (2024). Novel Insights into the GCN2 Pathway and Its Targeting. Therapeutic Value in Cancer and Lessons from Lung Fibrosis Development. FEBS J..

[108] Zhang X., Yao J., Li X., Niu N., Liu Y., Hajek R.A., Peng G., Westin S., Sood A.K., Liu J. (2023). Targeting Polyploid Giant Cancer Cells Potentiates a Therapeutic Response and Overcomes Resistance to PARP Inhibitors in Ovarian Cancer. Sci. Adv..

